# Rho Family of Ras-Like GTPases in Early-Branching Animals

**DOI:** 10.3390/cells9102279

**Published:** 2020-10-13

**Authors:** Silvestar Beljan, Maja Herak Bosnar, Helena Ćetković

**Affiliations:** 1 Division of Molecular Biology, Ruđer Bošković Institute, HR-10000 Zagreb, Croatia; silvestar.beljan@biol.pmf.hr; 2 Division of Molecular Biology, Faculty of Science, University of Zagreb, HR-10000 Zagreb, Croatia; 3 Division of Molecular Medicine, Ruđer Bošković Institute, HR-10000 Zagreb, Croatia; mherak@irb.hr

**Keywords:** non-bilaterian animals, Rho GTPases, Cdc24, Rho, Rac, RhoBTB, Miro, Porifera, Ctenophora, Placozoa, Cnidaria

## Abstract

Non-bilaterian animals consist of four phyla; Porifera, Cnidaria, Ctenophora, and Placozoa. These early-diverging animals are crucial for understanding the evolution of the entire animal lineage. The Rho family of proteins make up a major branch of the Ras superfamily of small GTPases, which function as key molecular switches that play important roles in converting and amplifying external signals into cellular responses. This review represents a compilation of the current knowledge on Rho-family GTPases in non-bilaterian animals, the available experimental data about their biochemical characteristics and functions, as well as original bioinformatics analysis, in order to gain a general insight into the evolutionary history of Rho-family GTPases in simple animals.

## 1. Introduction

The development of multicellular organisms depends on the ability of cells to detect and respond adequately to external signals, expressed by other cells. The intercellular signaling in embryonic development mediated by adhesion molecules, extracellular matrix, cytokines, morphogens, growth factors, or hormones has been extensively studied. Cell signaling is initiated by binding of ligands to their specific cell surface receptors, which are conversely converted into responses leading to gene transcription, cell shape modeling, adhesion, motility, and endo/exocytosis [1]. Although eukaryotic cells probably use hundreds of GTPases to control different processes, the members of the Ras superfamily have emerged as key players in the regulation of many important biological processes including growth and differentiation, morphogenesis, cell division and motility, cytokinesis, and trafficking through the Golgi, nucleus, and endosomes [2]. Small GTPases are low-molecular-weight (Mr of 20–25 kDa) monomeric guanine nucleotide-binding proteins. They display a conserved structural backbone of 5 G-boxes involved in GTP-binding and GTPase activity [3]. The Ras-related small GTPases are divided into five subfamilies: Ras, Rho, Arf, Rab, and Ran. Ras family members are activated by diverse extracellular stimuli that trigger a series of intracellular signaling events. This cascade of events eventually controls gene transcription, which leads to activation of fundamental cellular processes, including cell growth and differentiation. Ras proteins, the first small GTPases discovered [4], regulate cell growth, proliferation, and differentiation [5,6]. Rho proteins (Rho, Rac, and Cdc42) control the assembly and organization of the actin cytoskeleton, which vastly influences cell morphology [7], while Rab and Arf represent key regulators of secretory and endocytic pathways during vesicle trafficking and microtubule dynamics [8,9]. Ran is the last discovered GTPase and has a central role in the translocation of RNA and proteins through the nuclear pore complex [10]. Numerous studies suggest a very complex functional diversity of the Ras protein superfamily. Their conserved G-domain structural backbone involved in GTP-binding and GTPase activity enables common biochemical properties, while each of them recognizes its individual binding partners [6,11]. The diversity of the Ras superfamily is demonstrated by the multiplicity of their upstream regulators and downstream target proteins to which the Ras-related small GTPases bind [12,13].

### 1.1. The Rho Family of Proteins—Rho GTPases

**Figure 1:**
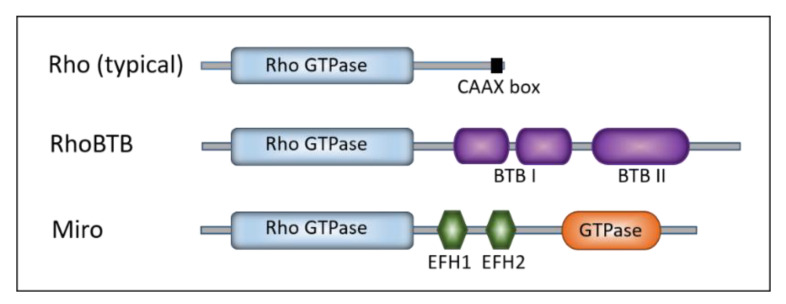
Schematic representation of Rho GTPases. Colored boxes represent characteristic structures: the Rho GTPase domain (light blue), CAAX box (black), BTB (broad complex/tramtrack/bric-a-brac) domain I and II (violet), EFH (EF-hand calcium binding) domain 1 and 2 (green), and the second GTPase domain in Miro (orange).

Rho GTPases are small G proteins found in all eukaryotes [14]. Rho family members are divided into nine subfamilies (Rho, Rac, Cdc42, RhoDF, Rnd, RhoUV, RhoH, RhoBTB, and Miro) according to their similar but not identical properties, such as primary amino acid sequence, structural motifs and biological functions. Similar to other Ras-like GTPases, Rho GTPases are active when bound to GTP and inactive when bound to GDP. Once activated, they bind effectors that mediate the cellular response [3]. The Rho signaling module is a complex regulatory network that includes over 240 proteins in human [15]. The Rho-family members are defined by the presence of a Rho-specific insert located between the G4 and the G5 boxes, involved in binding to effectors and regulators [16,17,18]. Typical Rho proteins are usually low-molecular-weight proteins and consist only of the GTPase domain (conserved structural backbone of 5 G-boxes) and short N- and C-terminal extensions (Figure 1).

**Figure 2:**
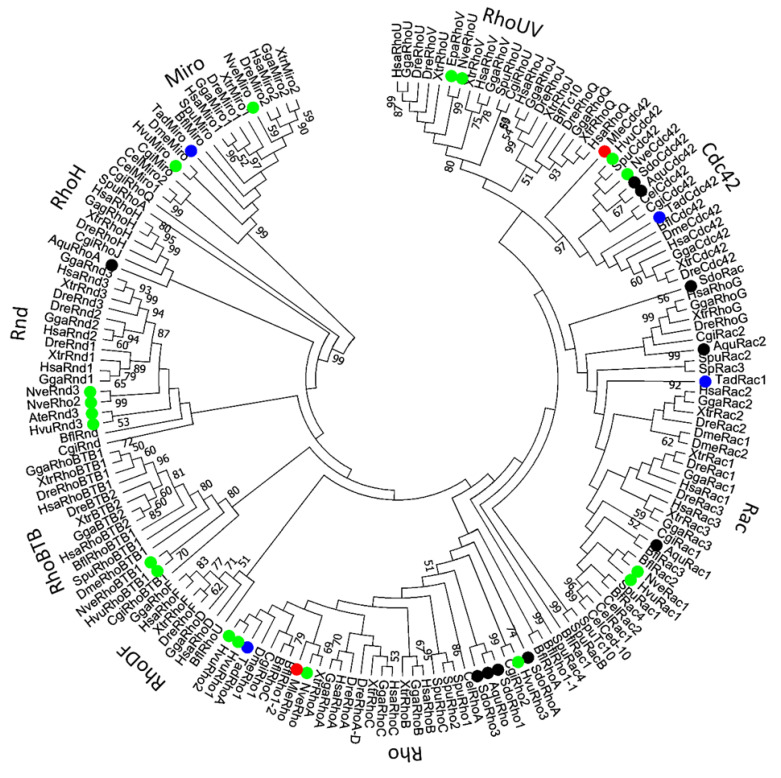
Phylogenetic tree of Rho GTPases in Metazoa. The evolutionary history of Rho GTPases from selected metazoan species (accession numbers are presented in Table S1.) was inferred using the Maximum Likelihood (ML) method based on the JTT matrix-based model [28]. Evolutionary analyses were conducted in MEGA7 [29]. The topological stability of the maximum likelihood (ML) tree was evaluated by 1000 bootstrapping replications. The bootstrapping values higher than 50 are indicated by numbers at the nodes. Rho GTPases from Cnidaria are marked in green, Porifera in black, Placozoa in blue, and Ctenophora in red.

Within their GTPase domains, Rho family members show approximately 30% amino acid identity with other Ras proteins and 40–95% identity within the Rho family [7]. The human genome encodes 22 Rho GTPases (Table 1) [19]. The majority of the functional information on the Rho family came from studies of typical Rho GTPases: RhoA, Rac1, and Cdc42 [7]. All three of them regulate actin dynamics and cytoskeleton reorganization that effect cell shape and motility [20,21]. Members of the Rho family power the reorganization of the actin cytoskeleton as a response to external stimuli, affecting a broad array of cellular processes and characteristics such as adhesion, migration, proliferation, shape, permeability, and polarity [15]. The members of the RhoBTB subfamily display additional atypical features: they are much larger than the typical GTPases, and they have additional domains [22]. The RhoBTB has two so-called broad complex/tramtrack/bric-a-brac (BTB) domains found to mediate homomeric or heteromeric protein–protein interactions (Figure 1). The RhoBTB functions as a tumor suppressor [22]. The members of the Miro subfamily were, at first, grouped into the Rho family on the basis of the similarity of their N-terminal GTPase domains (Figure 1) [23]. Later, they were found to be closely related, but likely a distinct Ras-like family [14]. The Miro GTPases localize within mitochondria and might promote apoptosis [23]. The presence of Rho family members in all eukaryotic supergroups indicates that the genes encoding Rho GTPase were present in the last eukaryotic common ancestor (around 1.4–1.8 billion years ago) [24,25]. Global evolutionary analyses of the Rho family indicate that the phylogenetic relationships among Rho GTPases are not strongly supported [14,26]. Namely, the Rho GTPase subfamily is more closely related to Rac than to other Rho subfamilies. Unicellular eukaryotes, fungi, and ancestral Metazoa contain only Rac, Cdc42, and RhoA GTPases (Table 1) [15]. The fact that Rho genes are absent in sub-clades of Chlorophyta, Trypanosomatidae, Stramenopiles, and Alveolates can be explained by multiple independent gene-loss events [27]. The Rho family expanded in Metazoa (around 700 million years ago), probably as a result of duplication events and lateral gene transfers [15] (Table 1 and Figure 2).

### 1.2. Non-Bilaterian Animals

The non-bilaterians include four animal phyla: Porifera (sponges), Cnidaria, Ctenophora (comb jellies), and Placozoa. These lineages branched off at the base of the animal tree of life before the origin of Bilateria and are thus referred to as early-branching animals (also known as non-bilaterian animals, “basal metazoans”, or “simple (early)” animals). These four phyla are crucial for understanding the evolution of animals. Fossil findings from Ediacaran assemblages (580 million years (Myr) old) suggest that the first metazoan organisms were diploblastic, similar to modern sponges and cnidarians [30]. Cnidaria and Ctenophora are primarily radially symmetric (Radiata) [31], which distinguishes them from other metazoans with two body axes (Bilateria). According to paleontological records from the Burgess Shale assemblages, the bilaterian diversification occurred within a short period in the lower Cambrium [32]. Deep metazoan phylogeny is difficult to resolve and many questions regarding the origin and early evolution of animals remain unanswered [33]. Major characteristics of all four basal metazoan groups are generally the lack of bilateral symmetry in their adult body plans and different developmental programs compared to other animals. Most sponges are asymmetrical or, less often, radially symmetrical. Cnidarians are radially symmetrical, while placozoans lack symmetry. Most ctenophoran species have modified radial (biradial) symmetry [34,35]. On the contrary, almost all Bilateria are triploblastic and bilaterally symmetrical. The status of Porifera (sponges) is unclear, since some authors claim that they have no germ layers. Sponges have been described as organisms devoid of true epithelia, lacking the gastrulation stage during development [36,37]. The other three non-bilaterian phyla are diploblasts, their adult tissues are derivatives of two primary germ layers: the ectoderm and the endoderm (endomesoderm) [34]. To this date, genomes of few non-bilaterian species have been published: sponge *Amphimedon queenslandica* [38], ctenophore *Mnemiopsis leidyi* [39], placozoan *Trichoplax adhaerens* [40], and cnidarians *Hydra vulgaris* [41] and *Nematostella vectensis* [42]. In addition, a few draft genomes are available: cnidarians *Actinia tenebrosa* [43], *Montipora capitata* [44], *Actinia equine* [45], *Exaiptasia pallida* [46], *Pocillopora damicornis* [47], *Alatina alata* [48], *Stylophora pistillata* [49], *Acropora digitifera* [50], *Dendronephthya gigantea* [51], *Acropora millepora* [52], and ctenophore *Pleurobrachia bachei* [53].

#### 1.2.1. Sponges

Sponges (phylum Porifera) are ancient animals, one of the earliest branching animal phylum that changed little in the last 800 million years [54]. Therefore, they are a remarkable model for studying the origin and the early evolution of Metazoa. Sessile as adults, sponges lack true tissues and organs as well as any recognizable sensory or nervous structures. They also lack a clear anterior–posterior (AP) polarity as adults, but the larvae do swim directionally. Their body plan is adapted for filtering water to enable feeding and respiration [36]. The sponge body is made of three layers. Polygonal cells called *pinacocytes* cover the outside of the sponge. The inside layer is built of *choanocytes*—cells with a flagellum surrounded by a collar. The middle layer called *mesohyl* is a matrix of glycoproteins with several types of motile cells and skeletal elements (calcareous or siliceous *spicules* and/or protein *spongin*). Bigger and more complex sponges usually have additional structural features, such as specialized *choanocyte* chambers and a network of water channels, but the basic body structure is always conserved. Sponge bodies are covered by numerous small openings (pores) called *ostia* and have at least one big opening—*osculum*. Due to the coordinated action of choanocytes, water carrying food and oxygen enters the sponge body through ostia, moves through the channels and chambers, and exits the sponge through the osculum. Besides producing the water current, the choanocytes filter food particles, which are subsequently digested by the cells in the mesohyl. Currently, there are more than 9000 valid sponge species inhabiting diverse marine and freshwater habitats [35]. There are four classes of sponges: *Demospongia*, *Hexactinellinda*, *Calcarea*, and *Homnoscleromorpha*. In contrast to their simple morphology, sponges have strikingly complex genomes and many of their genes are highly similar to their vertebrate homologs [38]. Therefore, they provide the best possible insight into the metazoan last common ancestors’ genome and proteome features [38,55,56]. The main poriferan evolutionary developmental biology models are the demosponge *A. queenslandica* and the calcarean *Sycon ciliatum* in which several steps of development have been investigated and genomes and transcriptomes sequenced [34]. Sponges represent an important model for studying ancestral metazoan homologs and their features before the diversification and specialization of these genes in higher animals. Sponges possess most of the genes present in animals with true tissues (*Eumetazoa*), numerous genes involved in early development, and carcinogenesis, as well as totipotent cells.

#### 1.2.2. Placozoans

Placozoans are a group with the simplest morphology among the metazoans. Their embryology is still unknown. Their disc-shaped bodies, without a gastric cavity, consist of two epithelial layers with a third layer of loosely arranged fiber cells between them. The lower of the two layers, which is in contact with the substrate, has specialized ciliated and gland cells used for adherence and feeding and enables the animal to move by crawling [40]. The placozoans possess baso-apical polarity, they have no mesenchymal tissues and are composed of six somatic cell types. Placozoans are tiny animals, usually less than two millimeters in diameter, without specialized muscle or nervous structures [35]. For a long time, only a single placozoan species was known—*Trichoplax adherens*, but lately, it has been discovered that the phylum is much more diverse [57,58]. The *Trichoplax* genome is compact, but with many genes involved in complex cellular processes found in “higher” animals. The genome contains 11,514 protein-coding genes that are closely related to those of cnidarians [40]. Placozoans and sponges are the only animals that do not possess true tissues and are thus sometimes grouped together into Parazoa [35].

#### 1.2.3. Cnidarians

Cnidarians are characterized by an archetypal gastrula-shaped body plan. They possess the basic three-layered body structure consisting of an outside and an inside epithelium with a gelatinous mesoglea in the middle. The cnidarian body reveals crucial evolutionary innovations: basement membrane, muscle cells, simple nervous system, and sensory organs. Cnidarians possess cnidocytes—specialized explosive cells with harpoon-like structures and toxic content designated to capture prey and/or as a defense mechanism against predators. Cnidarians appear in two significantly different forms: a free-swimming medusa (jellyfish), or a sessile polyp, attached to the substrate, sometimes connected with others, forming a colony. Both forms display radial symmetry with tentacles surrounding the mouth [35]. There are over 11,000 described species of cnidarians divided into five classes: *Hydrozoa*, *Scyphozoa*, *Cubozoa*, *Staurozoa*, and *Anthozoa*. [59]. Cnidarians are well-known for their ability to regenerate lost parts. Hydra has been used for decades as a model organism for studying regeneration processes in metazoans. Regeneration in these organisms occurs by a mechanism that does not require either proliferation or growth (morphalaxis) [60,61]. The genomes of cnidarians such as the sea anemone *N. vectensis* [42] or the freshwater polyp *Hydra* [41] reveal a repertoire of about 18,000 protein-coding genes, indicating the genomic complexity of the common bilaterian–cnidarian ancestor [41,42].

#### 1.2.4. Ctenophores

The ctenophores or comb jellies have a body structure identical to cnidarians—an outside and an inside epithelium with mesoglea between them. However, there are several important differences. Ctenophores do not have cnidocytes, and their epithelia have two cell layers. The multiciliated cells typical for this group usually form eight rows (combs) along the body, making ctenophores the largest organisms that swim by ciliary motion. Same as cnidarians, ctenophores have muscle cells and a simple nervous system with sensory organs. Ctenophores and cnidarians, as well as all Bilateria, developed tissues and, therefore, belong to the supergroup Eumetazoa. Ctenophoran species are divided into two classes: *Nuda* and *Tentaculata*, which have long tentacles with sticky cells used to capture pray [35]. Evolutionary analyses show that many of the ctenophoran orders, families, and even genera are not monophyletic [62]. Analyses of 18S RNA and ITS1 + 5.8S + ITS2 sequences of several species of this phylum suggest that today’s ctenophores have passed through a relatively recent radiation followed by a bottleneck [62,63]. The main ctenophore models used in evolutionary developmental biology (evo–devo) are the invasive species *Mnemiopsis leidyi* [39] and *Pleurobrachia pileus* [64].

### 1.3. Phylogenetic Relationship of Non-Bilaterian Animals

**Figure 3:**
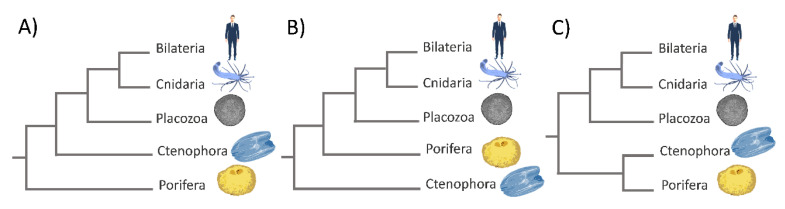
Phylogenetic relationships among non-bilaterian phyla and their relationship with Bilateria. (**A**) Porifera sister to all other metazoan; (**B**) Ctenophora sister to all other metazoan; (**C**) Porifera + Ctenophora clade sister to all other Metazoa.

One of the most disputed issues concerning the phylogeny of animals is the phylogenetic relationships among non-bilaterian phyla (Porifera, Cnidaria, Ctenophora, and Placozoa) and their relationship with Bilateria [65,66]. Understanding the relationships among phyla and the true topology of the metazoan tree of life is crucial for answering questions concerning the emergence of metazoan characteristics and their evolution during the last 700 million years [66,67]. The traditional view is that sponges are the earliest-branching lineage, while placozoans are a sister group of Eumetazoa (Figure 3A) [68].

Phylogenomic studies based on the genomic sequences of the sponge *A. queenslandica*, the placozoan *T. adherens*, and the cnidarian *N. vectensis* are in agreement with this traditional view [38,40,42]. Other studies placed ctenophores at the base of the animal tree of life (Figure 3B), which was, in turn, challenged by other studies.The criticism was addressed towards the phylogenetic methods used, which could have influenced the results [31,69]. Another study placed Porifera + Ctenophora as the earliest-branching animals (Figure 3C) [39]. This finding could have a huge impact on our understanding of early metazoan evolution. It would mean that the last common ancestor of Metazoa was quite complex, while sponges became simpler as an adaptation to their highly specialized lifestyle as sessile water filterers. Even though the relationship of the basal metazoans is still controversial, multiple lines of evidence suggest that early animals possessed a poriferan-like body plan, and thus, extant sponges may provide key knowledge on the origins of complex animal bodies [70]. Although sponges are structurally simple organisms, their molecular machinery is similar to the ones in other more complex animals. Furthermore, they possess genes that may be used in pathways for crucial physiological events, such as growth, differentiation, cell specialization, adhesion, and sensory functions [36]. In order to resolve relationships within basal metazoan phyla, we need to increase the phylogenomic sampling, improve the phylogenomic tree construction methods, and connect molecular methods with classical biology. The exponential increase in research publications in the field of evo–devo biology has put non-bilaterian animals again in the spotlight of investigation. The use of simple models contributed especially to the identification of pathways highly conserved in most eukaryotes. The molecular studies of simple (early branching) animals provide important information about the origin of the main signal pathways common to all animals. Herein, we review the current knowledge on Rho-family GTPases in non-bilaterian animals, the available experimental data about their biochemical characteristics and functions as well as original bioinformatics analysis, in order to gain a general insight into the evolutionary history of Rho-family GTPases in simple animals.

## 2. The Rho Family of Proteins, Rho GTPases, in Non-Bilaterian Animals

The repertoire of Rho genes/proteins remains quite similar in terms of number and complexity from unicellular eukaryotes to non-bilaterian animals [14]. Rho families mainly arose through the duplication of Rho or Rac genes. Rho family members are divided into nine subfamilies; Rho, Rac, Cdc42, RhoDF, Rnd, RhoUV, RhoH, RhoBTB, and Miro. The evolutionary analysis of the Rho family indicate that Rac protein is probably the founder of the Rho family [14]. The ancestral Rac duplications in metazoans were associated with early specialization, leading to Cdc42 in charge of controlling cell polarity and Rho responsible for cytokinesis [71,72]. Rho, Cdc42, and RhoBTB probably emerged from Rac within the 100–200 million years (Myr) period [73]. Rho, Rac, Cdc42, and RhoBTB are present in all bilaterians, with the exception of RhoBTB, which is absent in *C. elegans* and *O. dioica* [14]. This confirms the well-documented roles of Rho, Rac, and Cdc42 in basic cellular metabolism and supports the data implicating RhoBTB2 in the control of proliferation, apoptosis, and membrane trafficking [74,75]. The time of emergence of Rnd and RhoUV is consistent with their roles in the acquisition of muscle and nerve cells, while the Cdc42 isoforms, RhoJQ and RhoDF, probably emerged at the time of origin of the vertebrate central nervous system [27]. A very important common characteristic of the entire Rho family is its high evolutionary dynamics, visible through the high incidence of gain and loss of its members in different animal lineages [14].

In this study, we systematically identified and characterized Rho family members from basal Metazoa (Table 2).

**Figure 4:**
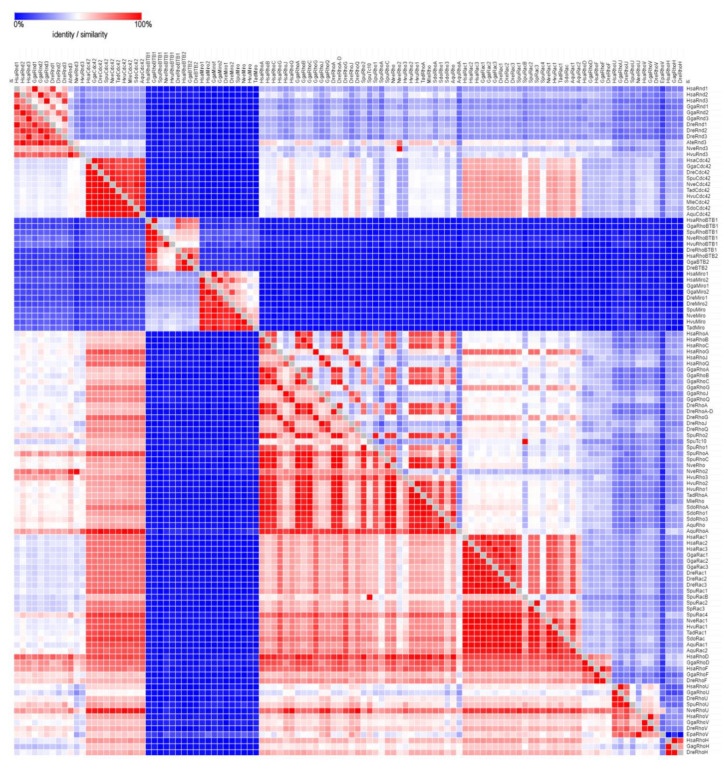
The summarized amino acid similarity of metazoan Rho proteins (MatGAT2.01 (Scoring Matrix BLOSUM62)) is presented using a heat map (red color indicates a high and blue a low percentage of sequence identity/similarity) generated by Morpheus (https://software.broadinstitute.org/morpheus). The detailed accession numbers of the protein sequences are shown in Table S1.

We searched the whole-genome sequences of basal Metazoa at the National Center for Biotechnology Information database (NCBI) using blastp algorithm (https://blast.ncbi.nlm.nih.gov/Blast.cgi) to identify Rho GTPase homologs in Porifera, Cnidaria, Ctenophora, and Placozoa, with bilaterian Rho protein sequences from NCBI as queries (Table 1 and Table S1). We aligned Rho family members from basal Metazoa and representatives of different bilaterian lineages using ClustalX [76]. Amino acid identity and similarity percentages matrices of metazoan Rho proteins were generated using MatGAT2.01 with BLOSUM62 scores [77]. The summarized identities of metazoan Rho proteins are illustrated using a heat map generated by Morpheus (https://software.broadinstitute.org/morpheus) (Figure 4).

**Figure 5:**
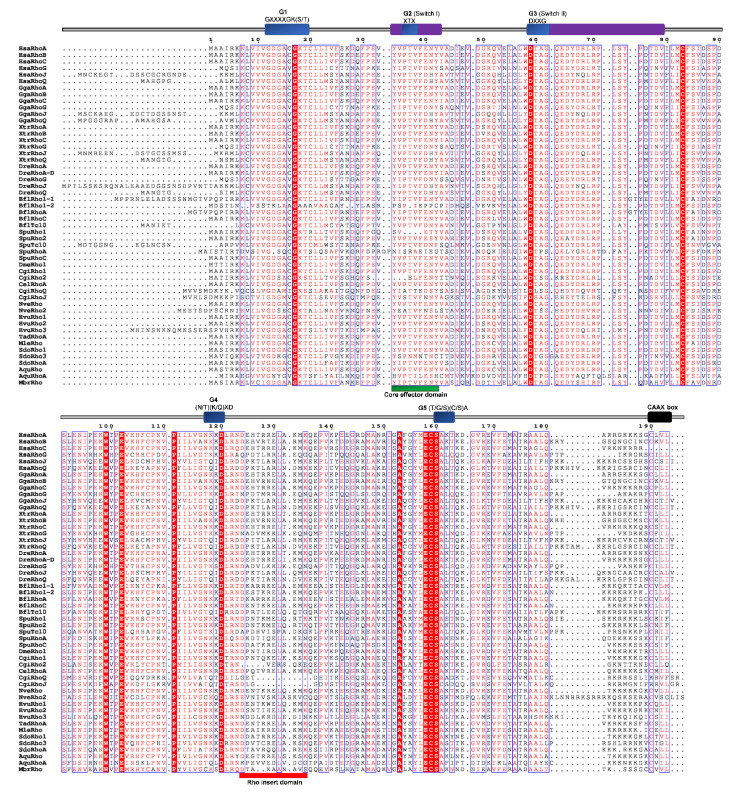
Sequence alignment of the Rho subfamily. The amino acid sequences of Rho GTPases were aligned using ClustalX, the secondary structures predicted using ESPript. Regions of the Rho proteins are indicated above the alignment; blue are the G domains, (G1, G2, G3, G4, and G5 with their respected motifs), purple are switch 1 and switch 2 domains, red is the Rho insert, green is the polybasic region, and black is the CAAX box. Blue frames are indicators of the conserved residues; white letters in red boxes represent strict identity, red letters in white boxes represent similarity.

**Figure 6:**
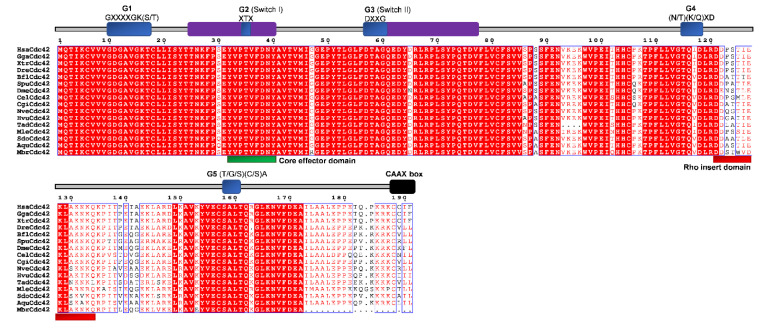
Sequence alignment of the Cdc42 subfamily. The amino acid sequences of Cdc42 GTPases were aligned using ClustalX, the secondary structures predicted using ESPript. Regions of the Cdc42 proteins are indicated above the alignment; blue are the G domains, (G1, G2, G3, G4, and G5 with their respected motifs), purple are switch 1 and switch 2 domains, red is the Rho insert, green is the polybasic region, and black is the CAAX box. Blue frames are indicators of the conserved residues; white letters in red boxes represent identity, red letters in white boxes represent similarity.

**Figure 7:**
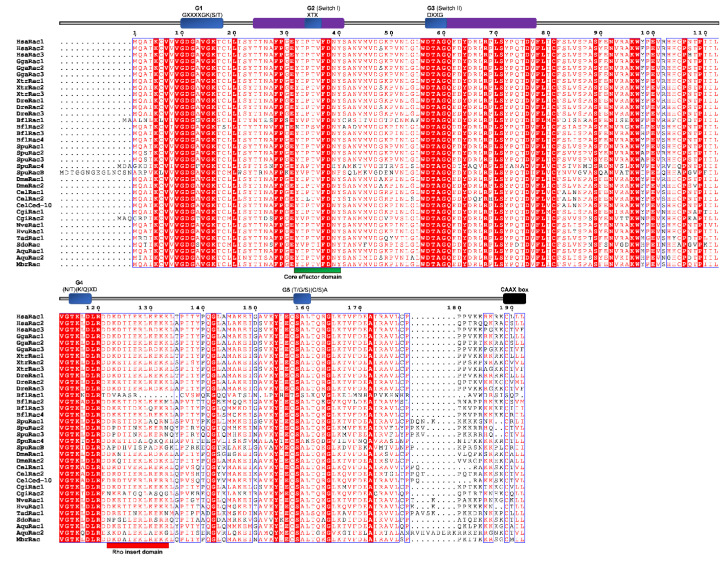
Sequence alignment of the Rac subfamily. The amino acid sequences of Rac GTPases were aligned using ClustalX, the secondary structures predicted using ESPript. Regions of the Rac proteins are indicated above the alignment; blue are the G domains, (G1, G2, G3, G4, and G5 with their respected motifs), purple are switch 1 and switch 2 domains, red is the Rho insert, green is the polybasic region, and black is the CAAX box. Blue frames are indicators of the conserved residues; white letters in red boxes represent identity, red letters in white boxes represent similarity.

**Figure 8:**
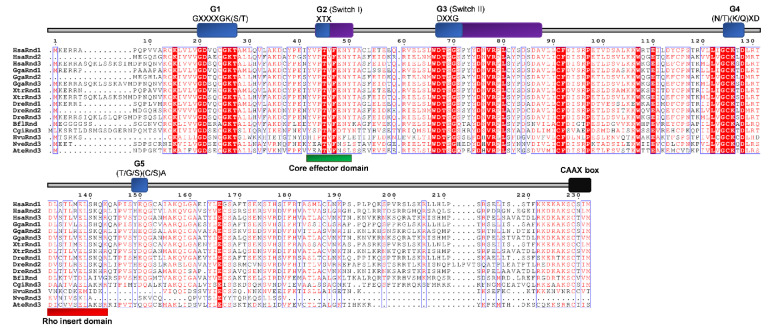
Sequence alignment of the Rnd subfamily. The amino acid sequences of Rnd GTPases were aligned using ClustalX, the secondary structures predicted using ESPript. Regions of the Rnd proteins are indicated above the alignment; blue are the G domains, (G1, G2, G3, G4, and G5 with their respected motifs), purple are switch 1 and switch 2 domains, red is the Rho insert, green is the core effector domain, and black is the CAAX box. Blue frames are indicators of the conserved residues; white letters in red boxes represent identity, and red letters in white boxes represent similarity.

**Figure 9:**
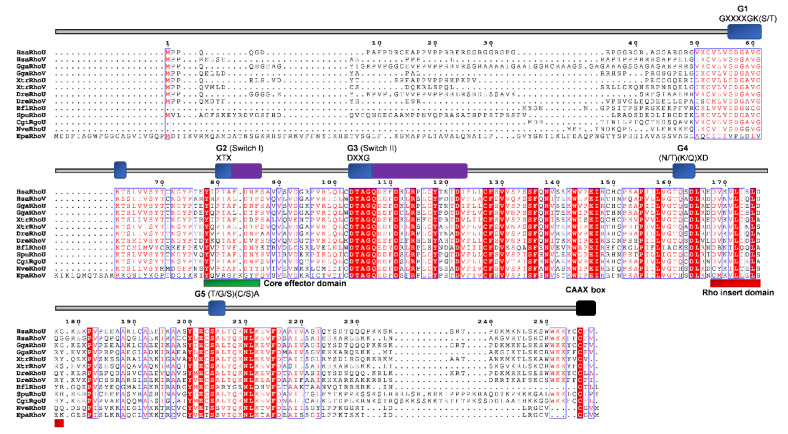
Sequence alignment of the RhoUV subfamily. The amino acid sequences of RhoUV GTPases were aligned using ClustalX, the secondary structures predicted using ESPript. Regions of the RhoUV proteins are indicated above the alignment; blue are the G domains, (G1, G2, G3, G4, and G5 with their respected motifs), purple are switch 1 and switch 2 domains, red is the Rho insert, green is the core effector domain, and black is the CAAX box. Blue frames are indicators of the conserved residues; white letters in red boxes represent identity, and red letters in white boxes represent similarity.

**Figure 10:**
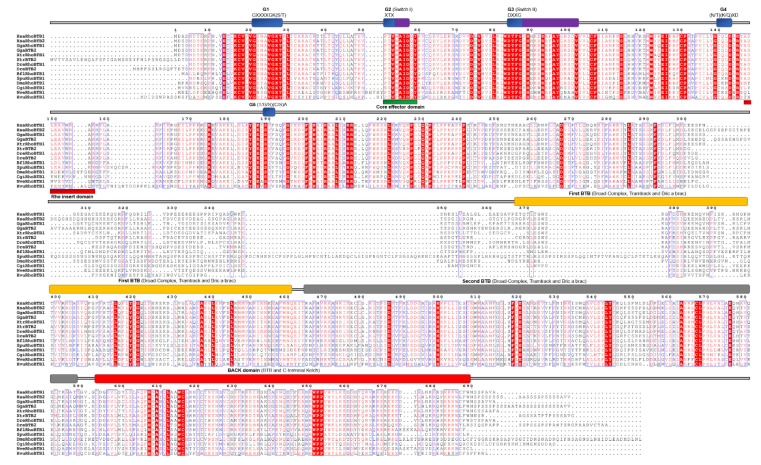
Sequence alignment of the RhoBTB subfamily. The amino acid sequences of RhoBTB GTPases were aligned using ClustalX, the secondary structures predicted using ESPript. Regions of the Rho proteins are indicated above the alignment; blue are the G domains, (G1, G2, G3, G4, and G5 with their respected motifs), purple are switch 1 and switch 2 domains, orange the first BTB domain, gray the second BTB domain, and red represents the C-terminus back domain. Blue frames are indicators of the conserved residues; white letters in red boxes represent identity, red letters in white boxes represent similarity.

Conserved domains and motifs were identified by simple modular architecture research tool prediction (SMART; http://smart.embl-heidelberg.de/) and further confirmed through sequence alignment (ClustalX) with Rho proteins generated by ESPript (http://espript.ibcp.fr/ESPript/ESPript/) (Figure 5, Figure 6, Figure 7, Figure 8, Figure 9 and Figure 10). We used human Rho proteins with a completely resolved protein architecture as reference sequences. In order to determine the phylogenetic relationships of the Rho homologs in the basal Metazoa and known Rho proteins from Mammalia, Aves, Amphibia, Chordata, Cephalochordata, Echinodermata, Arthropoda, Nematoda, and Mollusca, we constructed a phylogenetic tree using the maximum likelihood algorithm in the MEGA 7 (Figure 2) [29]. Our phylogenetic analysis confirmed that the Rho family of small GTPases can be divided into nine major subfamilies: Rho, Rac, Cdc42, Rnd, RhoDF, RhoUV, RhoH, RhoBTB, and Miro. The members of these subfamilies in basal Metazoa were categorized according to their phylogenetic clustering (Figure 2.).

### 2.1. Rho

Ćetković and coworkers [2] inspected 13,000 partial cDNA sequences (ESTs) from the marine sponge *Suberites domuncula* and identified cDNA sequences coding for three homologs in Metazoa, named SdRho1, SdRho2 (SdRhoA), SdRho3. All three proteins from *S. domuncula* have the Rho GTPase domain characteristic for Rho subfamily members and are responsible for nucleotide binding and hydrolysis (G1–5 boxes) and two regions that specifically bind regulators or effectors—the switch 1 and 2 regions. In addition, all of them have the Rho insert, the C-terminal CXXX motif (C: cysteine, A: aliphatic, X: any amino acid), which undergoes post-translational lipid modifications responsible for membrane targeting, and a polybasic region adjacent to the CAAX motif which contributes to association to membranes, interaction with regulators, and subcellular localization (Figure 5) [2].

Boureux and coworkers [14] analyzed a draft genome of the sponge *A. queenslandica* (Reniera sp. JGI-2005) and identified one Rho protein, while Fort states [27] that the genome of this sponge has three Rho s.s. GTPases (stricto sensu, i.e., the ancestor to human RhoA-C). We have identified two Rho proteins (AqRho and AqRhoA) in the genome of *A. queenslandica*. This is probably due to incomplete and/or low-quality genome assembly and gene annotation. HvRho1, HvRho2, and HvRho3 Rho GTPases are present in the cnidarian *H. vulgaris*, while NvRho and NvRho2 are present in *N. vectensis* (Table 1 and Figure 2) [14,78]. A varying number of paralogs in cnidarians is probably a consequence of lineage-specific duplications. We have identified a single Rho protein (*TaRho*) in the genome of the placozoan *T. adherens* and in the genome of the ctenophore *M. leidyi* (*MlRho*) (Table 1 and Figure 2).

We aligned Rho homologs from basal Metazoa and representatives of different bilaterian lineages (Figure 5), and analyzed protein sequence identity/similarity (Figure 4). The Rho domain (G1, G2 switch 1, G3 switch 2, G4, and G5), the Rho insert (10–15 residue) important for the regulation of Rho GTPases activity, the polybasic site and CAAX box are present in all basal metazoan homologs of the human Rho subfamily proteins. HsaRhoC does not possess the CAAX box, while in CgiRhoJ and CgiRho, the cysteine is substituted with valine in the CAAX box (Figure 5). Rho subfamily homologs from basal Metazoa show 50.5–94.3% sequence similarity with the human Rho GTPases. The highest homology (identity/similarity) with the human RhoA protein is as follows: NveRho homolog from the cnidarian *N. vectensis* 89%/94%, TadRhoA homolog from the placozoan *T. adherens* 88%/93%, HvuRho1 homolog from the cnidarian *H. vulgaris* 88%/94%, AquRho from the sponge *A. queenslandica* 85%/90%, and SdoRho1 from the sponge *S. domuncula* 86%/91% (Table 2).

### 2.2. Cdc42

A single *Cdc42* gene was found in the genome of the sponge *A. queenslandica* [14,27]. Analysis of the cDNA sequences (ESTs) from the marine sponge *Suberites domuncula* identified only one Cdc42 protein, named SdCdc42 [2]. In addition, a single Cdc42 Rho GTPase is present in the cnidarians *H. vulagaris* (HvCdc42) and *N. vectensis* (NvCdc42) (Table 1 and Figure 2) [78]. We identified only one Cdc42 protein (*TaCdc42*) in the genome of the placozoan *T. adherens* as well as a single Cdc42 homolog (*MlCdc42*) in the genome of the ctenophore *M. leidyi* (Table 1 and Figure 2). We aligned Cdc42 homologs from basal Metazoa and representatives of different bilaterian lineages (Figure 6) and analyzed the protein sequence identity/similarity (Figure 4).

As shown in Figure 6, all the protein domains (G1, G2 switch 1, G3 switch 2, G4, and G5), the Rho insert, the polybasic site, and CAAX box are present in basal metazoan homologs of the human Cdc42 protein. Homologs of the Cdc42 subfamily from basal Metazoa display very high homology (92–95% similarity) with the human Cdc42. The highest homology with the human Cdc42 protein display the NveCdc42 homolog from the cnidarian *N. vectensis* (identity/similarity; 91%/95%) and AqCdc42 homolog from the sponge *A. queenslandica* (identity/similarity; 90%/95%). The TadCdc42 homolog from Placozoa *T. adherens* shows 86%/92% and SdoCdc42 from *S. domuncula* 89%/94% identity/similarity (Table 2).

### 2.3. Rac

Boureux and coworkers [14] analyzed the draft genome of the sponge *A. queenslandica* (Reniera sp. JGI-2005) and identified five Rac proteins (RRac1–RRac5), while Fort states [27] that the sponge genome possesses only one Rac gene/protein. We identified two Rac proteins (AqRac1 and AqRac2) in the genome of *A. queenslandica*, while a single Rac protein, SdRac, was identified in the marine sponge *Suberites domuncula* EST database (Table 1 and Figure 2) [2]. The same has been suggested for cnidarians [27] although the genome of *H. vulgaris* has two Rac genes (*HvRac1* and *HvRac2*) while *N. vectensis* has only one (*NvRac*) [78]. A varying number of Rac paralogs in sponges and cnidarians is probably a consequence of lineage-specific duplications. We identified only one *Rac* gene (HvRac1) in the genome of *H. vulgaris.* We have also identified one *Rac* gene (*TaRac*) in the genome of the placozoan *T. adherens*, while the genome of the ctenophoran *M. leidyi* seems to lack Rac subfamily Rho GTPase members (Table 1 and Figure 2). We aligned Rac homologs from basal Metazoa and representatives of different bilaterian lineages (Figure 7) and analyzed the protein sequence identity/similarity (Figure 4).

As shown in Figure 7, all the Rac protein domains (G1, G2 switch 1, G3 switch 2, G4, and G5), the Rho insert, the polybasic site, and CAAX box are present in basal metazoan homologs of the human Rac proteins. Rac subfamily homologs from basal Metazoa display high homology (80–94% similarity) with human Rac proteins. The highest homology (identity/similarity) with the human Rac proteins are as follows: TadRac homolog from Placozoa *T. adherens* 80%/87%, HvuRac1 homolog from the cnidarian *H. vulgaris* 89%/92%, AquRac1 homolog from Porifera *A. queenslandica* 88%/94%, and SdoRac homolog from Porifera *S. domuncula* 70%/83% with the human HsaRac1 protein. NveRac1 homolog from the cnidarian *N. vectensis* shows the highest identity/similarity (89%/93%) with the human HsaRac2 protein (Table 2).

### 2.4. Rnd

The Rnd gene/protein most likely emerged before the divergence of Cnidaria [27]. *Rnd* genes have been identified in several genomes of Anthozoa (the sea anemones *N. vectensis, Acropora digitifera*, and *Exaiptasia pallida*) and Hydrozoa (*H. vulgaris*). Furthermore, the cnidarian Rnd protein has substitutions in its G3 box and is, therefore, atypical compared to other Rnd subfamily protein members. Consequently, the cnidarians Rnd is devoid of GTPase activity and remains active until it is degraded [27]. Cnidarians possess a neural net [34]. Rnd3 deficiency causes neuromuscular defects with a reduced number of motor neurons in mice [79]. Therefore, it is possible that Rnd has a vital function in the development of the neuronal network. Cnidaria could serve as a useful model for addressing the potential ancestral Rnd function in connection to the development and evolution of the nervous system. The timing of emergence of Rnd is consistent with its assigned role in the origin of nerve cells [27]. Several other publications report that Rnd appears exclusively in chordates [14]. We confirm the presence of Rnd subfamily homologs only in genomes of cnidarians *H. vulgaris*, *N. vectensis* (Table 1 and Figure 2), and the draft genome of *Actinia tenebrosa* (Figure 4 and Figure 8).

We aligned Rnd homologs from cnidarians and representatives of different bilaterian lineages (Figure 8) and analyzed the protein sequences’ identity/similarity (Table 2 and Figure 4). As shown in Figure 8, the members of the Rnd subfamily contain Rho the GTPase domain (G1, G2 switch 1, G3 switch 2, G4, and G5), the Rho insert and the CAAX box. The G4 domain contains a C instead of N/T in all Rnd proteins. The G5 domain is not conserved in many Rnd proteins. The G5 domain of the CgiRnd3 homolog from *C. gigas* contains an I instead of (T/G/C). The NveRnd3 homolog from *N. vectensis* contains a YYT motif instead of (T/G/C)(C/S)A in the G5 domain and an incomplete C-terminus. The G5 domain of the HsaRnd2 (*H. sapiens*), GgaRnd2 (*G. gallus*), DreRnd2 (*D. rerio*), CgiRnd3 (*C. gigas*), HvuRnd3 (*H. vulgaris*), and AteRnd3 (*A. tenebrosa*) contains a Serine instead of Alanine (Figure 8). The NveRnd homolog from the cnidarian *N. vectensis* shows 35%/50% identity/similarity and HvuRnd3 homolog from *H. vulgaris* 28%/46% with the human HsaRnd3. The AteRnd3 homolog from *A. tenebrosa* shows 42%/64% identity/similarity with the human HsaRnd2 protein (Table 2).

### 2.5. RhoUV

The RhoUV gene/protein most likely emerged before the divergence of Cnidaria [27]. *RhoUV* genes have been identified in genomes of the cnidarian *N. vectensis* and *E. pallida* [24]. Furthermore, the cnidarian RhoUV protein has substitutions in the G3 box and, therefore, lacks the GTPase activity [27]. Cnidarians, unlike Porifera, have smooth and striated muscles. In vertebrates, the RhoU protein is involved in the development and function of the heart [80,81]. Therefore, the cnidarian RhoUV proteins could be a good model for investigating the functions of the ancestral RhoUV GTPases related to muscle biology. The time of origin of the RhoUV is consistent with its role in the acquisition of muscle cells [27]. Some other publications report that members of the RhoUV subfamily cannot be detected in genomes of cnidarians [14,78]. We confirm the presence of RhoUV subfamily homologs only in genomes of cnidarians *N. vectensis* (Table 1 and Figure 2) and *E. pallida* (Figure 4 and Figure 9).

We aligned RhoUV homologs from cnidarians and representatives of different bilaterian lineages (Figure 9) and analyzed protein sequence identity/similarity (Figure 4). As shown in Figure 9, the members of the RhoUV subfamily contain the Rho GTPase domain (G1, G2 switch 1, G3 switch 2, G4, and G5), the Rho insert, and CAAX box (Figure 9). The G4 domain of NvRhoUV from the cnidarian *N. vectensis* and EpaRhoV from the cnidarian *E. pallida* contains the H amino acid instead of (K/Q) (Figure 9). The EpaRhoV homolog from *E. pallida* contains an amino acids motif VXXXXVKI instead of GXXXXGK(S/T) in the G1 domain, and an XRX motif instead of XTX in the G2 domain. The BflRhoU homolog (*B. floridae*) has amino acid C instead of N/T in the G4 domain. The NveRhoU homolog from the cnidarian *N. vectensis* shows 45%/59% identity/similarity with human HsaRhoV, while the EpaRhoV homolog from *E. pallida* shows 30%/44% identity/similarity with the human HsaRhoU (Table 2).

### 2.6. RhoBTB

RhoBTB proteins are atypical Rho GTPases [7]. These GTPases are structurally different from other Rho-family members and possess considerable additional sequences after the Rho GTPase domain. In the G4 domain, the C amino acid is present instead of N/T, while in the G5 domain, the (T/G/C)(C/S)A motif is replaced with S(V/I/S)(F/V/Y/L) (Figure 1 and Figure 7). The additional C-terminal sequences include a tandem repeat of BTB domains and lack C-terminal CAAX prenylation signals [7]. According to previous studies, cnidarians either have two RhoBTB proteins [27] or, in case of *H. magnipapillata* (HmRhoBTB) and *N. vectensis* (NvRhoBTB), only one *RhoBTB* gene per genome [14,78]. We have also identified only one RhoBTB homolog in genomes of cnidarians *H. vulgaris* (HvRhoBTB) and *N. vectensis* (NvRhoBTB). Members of the *RhoBTB* subfamily of Rho GTPases were not found in genomes of sponges, placozoans, and ctenophores. We aligned RhoBTB homologs from cnidarians and representatives of different bilaterian lineages (Figure 10) and analyzed protein sequence identity/similarity (Figure 4).

The NvRhoBTB homolog from *N. vectensis* shows 37/54% identity/similarity, while the HvRhoBTB1 homolog from *H. vulgaris* 33%/51% identity/similarity with human RhoBTB proteins (Table 2).

### 2.7. Miro

**Figure 11:**
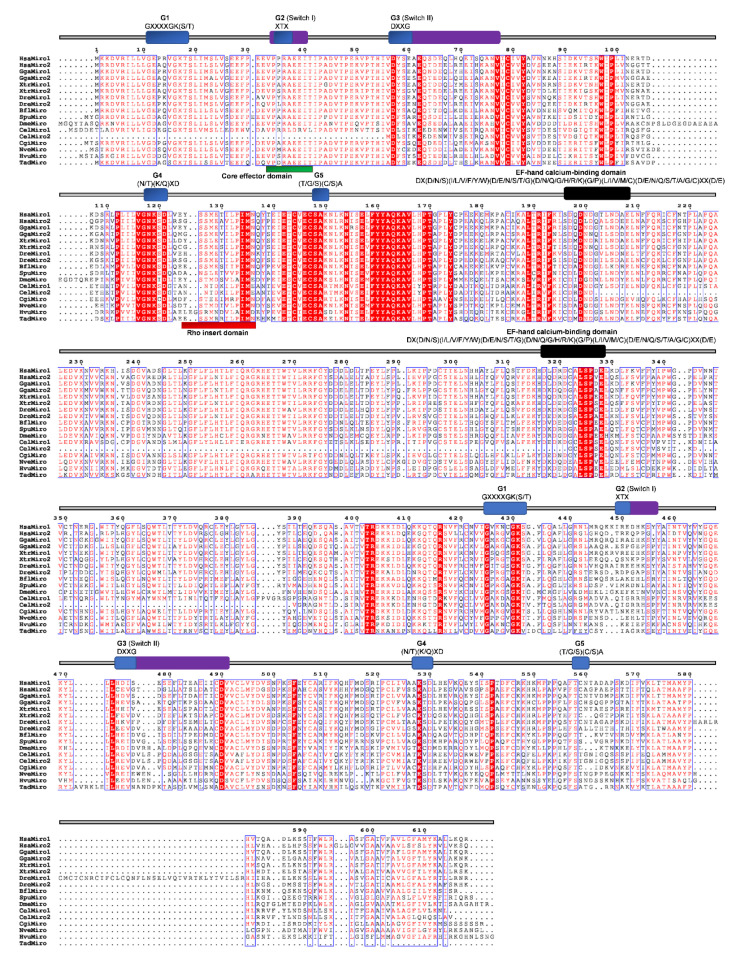
Sequence alignment of the Miro subfamily. The amino acid sequences of Miro GTPases were aligned using ClustalX, the secondary structures predicted using ESPript. Regions of the Miro proteins are indicated above the alignment; blue are the G domains (G1, G2, G3, G4, and G5) domains; purple represent the switch domains (I and II) and black the EF hand calcium binding domains with their respected motifs. Blue frames are indicators of the conserved residues; white letters in red boxes represent identity, red letters in white boxes represent similarity.

The Miro (mitochondrial Rho) GTPases are sometimes placed in the Rho family. However, this seems to be a separate, structurally unique, functionally specialized family of GTPases that most likely already existed in the last common eukaryotic ancestor [23,82]. The members of the Miro subfamily contain two putative GTPase domains and two EF-hand motifs but lack the Rho-specific insert sequence (Figure 1 and Figure 8). The N-terminus G3 domain and the C-terminus G-domains are not conserved. The C-terminus Rho-GTPase domain of Miro GTPases was previously considered to be a “relic“, but some studies observed its activity [83]. The first G3 domain contains the GXXE/A/I/S/E/Y motif instead of the DXXG, whereas only the XtrMiro2 possesses the DXXG motif. The second G1 domain of TadMiro from the placozoan *T. adherens* contains a Valine (V) instead of S/T. The second G2 domain is present only in the human HsaMiro2. The second G3 and G5 domains are not conserved (Figure 11). Cnidarians *H. vulgaris* (HvMiro) and *N. vectensis* (NvMiro) contain a single Miro subfamily member (Table 1 and Figure 2) [78]. Our searches did not find members of the Miro subfamily of Rho GTPases in sponge and ctenophore genomes. We aligned Miro homologs from basal Metazoa and representatives of different bilaterian lineages (Figure 11) and analyzed protein sequence identity/similarity (Figure 4). The NveMiro homolog from the cnidarian *N. vectensis* shows 53/70% identity/similarity, while the HvuMiro homolog from *H. vulgaris* shows 45%/65% identity/similarity with the human HsaMiro1 protein. The TadMiro from Placozoa *T. adherens* shows 50%/66% identity/similarity with the human HsaMiro1 protein (Table 2).

## 3. Rho GTPases in Non-Bilaterian Animals Participate in a Plethora of Signaling Pathways

### 3.1. Wnt Pathway

**Figure 12:**
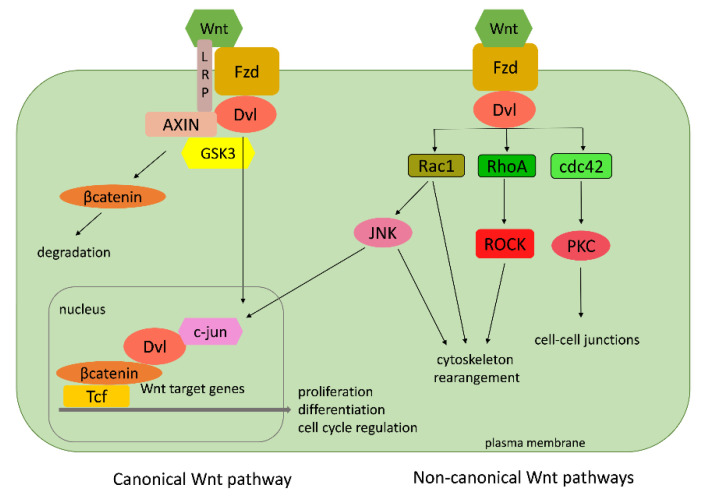
A simplified representation of the canonical and non-canonical Wnt signaling pathway. Small Rho GTPases are involved only in the non-canonical Wnt pathways. Wnt–Wnt family member; Fzd—Fizzled class receptor; Dvl—disheveled segment polarity protein; Rac1—Rac family small GTPase 1; Rho A—Ras homolog family member A; cdc42—cell division cycle 42; JNK—c-Jun N-terminal kinase; ROCK—Rho-associated coiled-coil containing protein kinase; PKC—protein kinase C; c-jun—Jun proto-oncogene; GSK3—glycogen synthase kinase 3; LRP—low-density-lipoprotein (LDL) receptor-related protein; Tcf—transcription factor.

Rho GTPases function as molecular switches in a vast number of signaling pathways. Some of those pathways can be found, usually in a simpler version, in basal metazoans as well. One of the most investigated is the Wnt pathway. The Wnt family comprises of secreted glycosylated proteins that influence cell growth, differentiation, and migration [84,85]. Wnt proteins act by binding to Frizzled (Fzd) receptors and thus activate two different signal transduction cascades: the canonical and the non-canonical (Figure 12). The canonical pathway includes the accumulation of β-catenin in the cytoplasm and its translocation into the nucleus, where is acts as a coactivator of transcription factors that belong to the Tcf/LEF (transcription factor/lymphoid enhancer-binding factor) family. The Rho GTPases take part in the non-canonical cascades, which lead to the rearrangement of the cell cytoskeleton causing changes in cell adhesion and migration properties (Figure 12) [86,87,88].

The non-canonical pathways include the planar cell polarity (PCP) pathway responsible for cell shape and the Wnt/calcium pathway, which controls the calcium in the cell. Neither of them include β-catenin. Contrary to the non-canonical Wnt pathways, the canonical Wnt signaling also requires single-span transmembrane proteins that belong to a subfamily of low-density-lipoprotein (LDL) receptor related proteins (LRPs): Lrp5 and Lrp6 in vertebrates, and their Drosophila ortholog (Arrow) [89]. The Wnt signaling pathway is an innovation of first multicellular animals (Metazoa), because a complete Wnt signaling pathway cannot be found in any of the single-cell organisms (protists). A core set of *Wnt* genes (a basal set of Wnt ligands, receptors, and cytoplasmic transducers) was already present in Porifera, Placozoa, and Ctenophora, while a complete functional repertoire of *Wnt* ligands is present only in Cnidaria. Their *Wnt* gene repertoire is simple compared to other metazoans. All existing *Wnt* genes are actively involved in gastrulation, pattern formation, and regeneration in non-bilaterian animals, providing a basic panel of tools for understanding the interchange of the canonical and two noncanonical pathways—the planar cell polarity (PCP) and the Wnt/calcium pathway [90].

The genomes of cnidarians the sea anemone *N. vectensis* (Putnam et al., 2007) and the freshwater polyp *H. vulgaris* [41] possess all bilaterian *Wnt* gene subfamilies, but some of the members within subfamilies are missing [90,91]. For instance, *Wnt-9* cannot be found in *N. vectensis* [92,93], whereas *H. vulgaris* lacks *Wnt4*, *-6*, and *-A*. The cnidarians possessing the complete repertoire of *Wnt* gene subfamilies suggests that the common (eumetazoan) ancestor of cnidarians and bilaterians already possessed a full-scale repertoire of *Wnt* gene ligands [92]. Cnidarian Wnts act in the canonical and in the planar cell polarity (PCP) pathways. Homologs of the PCP signaling pathway, including RhoA, ROCK-2, and Rac1, are present in cnidarians [90,93,94].

Adell and coworkers [95] described a Wnt-related gene, Sd-Frizzled, from Porifera. They also reported the isolation and phylogenetic characterization of several Wnt pathway-related genes from the demosponge *S. domuncula* [96]. Furthermore, they have found sponge homologs of small Rho GTPases, *RhoA* (*SdRhoA*) and *Cdc42* (*SdCdc42*), and their effector, myosin regulatory light chain (*Sdmrlc*). The isolation of a secreted frizzled related protein sFRP from another sponge species (*Lubomirskia baicalensis*) is reported [96]. The first genome analysis of the demosponge *A. queenslandica* [38] and the analysis of *S. domuncula* ESTs [56] enlightened the origin and evolution of the Wnt signaling pathway [97,98]. The sponge genome shows substantial conservation of gene families with cnidarians and bilaterian animals [40]. The main components of the canonical Wnt/β-catenin pathway are present, but those of the noncanonical pathways are missing [97,98]. *A. queenslandica* contains three, while the homoscleromorph sponge *Oscarella* has two *Wnt* genes, which are difficult to classify [38,98]. The growing accumulation of genomic and transcriptomic databases of early branched phyla has significantly increased our knowledge of the ancestral metazoan molecular toolkit. However, their involvement and function in specific molecular pathways remain to be elucidated, especially in Porifera. Adell et al. [96] analyzed the expression levels of small GTPases, *Sd-RhoA* and *Sd-Cdc42*, and the effector, *Sdmrlc*, involved in the non-canonical Wnt signaling in sponge cell cultures (sponge tissue, dissociated cells, adherent cells, and primmorphs (sponge cell aggregates)). All three genes were overexpressed in cultured cells compared to sponge tissue and adherent aggregates (primmorphs). These results suggest that the Wnt signaling in the sponge could be involved in the establishment of cell–cell contacts, and it is probably important for the regulation of cell–cell and cell–matrix interactions [96]. The placozoan *T. adhaerens* has three unclassified *Wnt* genes and the main components of the canonical Wnt signaling (Dvl (disheveled), Fzd, GSK3 (GSK3—glycogen synthase kinase 3), AXIN, β-catenin), but no Wnt antagonists [40]. In the ctenophore *M. leidyi*, orthologs of the key components of Wnt signaling have also been described [99].

### 3.2. Rho/Rock Pathway

**Figure 13:**
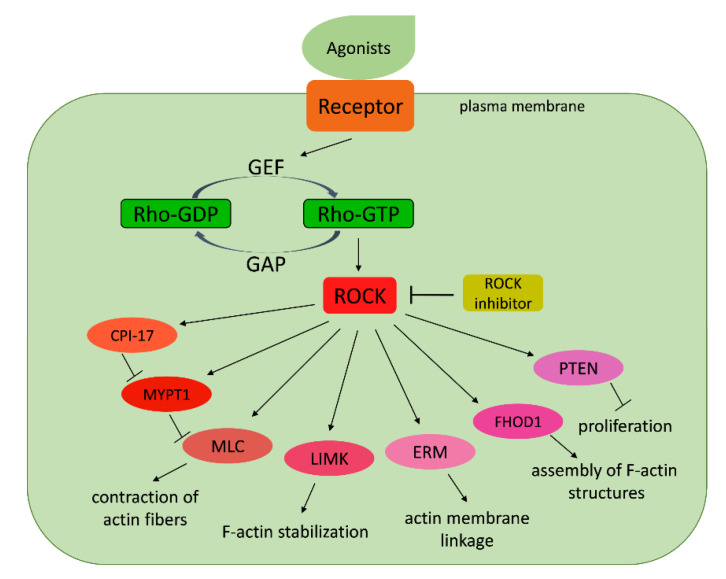
Simplified representation of the Rho/ROCK signaling pathway. GEF—guanine nucleotide exchange factor; GAP—GTPase activating protein; Rho—Ras homolog family member (activated if linked to GTP); ROCK—Rho-associated coiled-coil containing protein kinase; CPI-17—protein phosphatase 1 regulatory inhibitor subunit 14A; MYPT1—myosin phosphatase target subunit 1; MLC—myosin light-chain kinase; LIMK—LIM domain kinase; ERM—acronym from ezrin, radixin, moesin proteins; FHOD1—formin homology 2 domain containing 1; PTEN—phosphatase and tensin homolog.

The Rho associated coiled-coil protein kinase (ROCK) plays several important roles in development across bilaterian animal species. The Rho/ROCK signaling pathway affects cytoskeletal dynamics thus affecting cell shape, cell adhesion, and migration (Figure 13).

It is also involved in non-canonical Wnt signaling in bilateral animals. Consequently, the Rho/ROCK pathway is required for the induction of epithelial morphogenesis and establishing specific body plans in bilaterians. This highly conserved signaling pathway is thus the key for understanding molecular mechanisms that are probably involved in controlling early development of early-branching metazoans [100,101]. Schenkelaars and coworkers [102] found that sponges possess crucial proteins of the Rho/ROCK pathway, implying that these proteins were present in the last common ancestor of Metazoa. The structural domain analyses of the sponge homologs suggest that protein interactions described in bilaterians have likely already been present in sponges and conserved to vertebrates. Furthermore, the same author found that the sponge Em-ROCK kinase domain shows Rho kinase activity in vitro and that commercially available ROCK inhibitors target the sponge Em-ROCK protein. This finding supports the hypothesis that the Rho/ROCK pathway is an evolutionarily conserved module in animals. In vivo assays using ROCK inhibitors applied during early development of freshwater sponges provide solid evidence that ROCK is important for inducing the processes of morphogenesis and setting up the body plan [102]. Recently published data [103] suggest an essential role of FGFR (fibroblast growth factor receptor) and Rho-ROCK-myosin II pathways in the control of cell shape changes required for bud detachment in hydra. Using gene expression analysis and pharmacological inhibition, authors recognized a candidate signaling pathway through Rho, ROCK, and myosin II, which controls rearrangement of the actin cytoskeleton and bud base constriction. Inhibition of FGFR, Rho, ROCK, or myosin II kinase activity is permissive for budding but represses myosin phosphorylation, rearrangement of F-actin, and constriction. The young polyp remains permanently connected to the parent by a broad tissue bridge [103].

### 3.3. RhoGEFs and RhoGAPs

Rho proteins typically cycle between an inactive GDP-bound form and an active GTP-bound form. Rho GTPases are controlled by more than a hundred guanine nucleotide exchange factors (RhoGEFs) and GTPase-activating proteins (RhoGAPs). Their activity cycle is initiated by RhoGEFs and terminated by RhoGAPs. A vast number of regulatory proteins, RhoGEFs and RhoGAPs, significantly outnumber the 10 Rho family GTPase switch proteins they regulate, therefore enabling the precise control of Rho signaling specificity [104]. However, it should be noted that some Rho family GTPases do not behave like conventional Rho proteins in respect to their activation, e.g., Rnd. Rnds do not catalyze GTP hydrolysis and, therefore, do not require GEFs for activation [105].

More than 70 different RhoGAPs have been characterized in eukaryotes. The human genome encodes between 59 and 70 proteins containing the RhoGAP domain [106]. A comprehensive comparative analysis on RhoGAPs that would include basal metazoans has not been done so far. A comparative analysis of metastasis suppressors in Metazoa has revealed that homologs of Rho GTPase-activating protein DLC-1 (deleted in liver cancer 1) were present in all basal Metazoa (non-Bilateria) [107]. In another study, it was shown that the homologs of Rho GTPase-activating protein RhoGAP, ARHGAP11A (named MP-GAP for M Phase GAP) were present in Cnidaria and Placozoa [108].

The human genome encodes 82 different RhoGEFs. Dbl-like (diffuse B-cell lymphoma) members of the RhoGEFs form the largest family. Comparative analysis performed by [15] showed that the human Dbl-like family is composed of 71 members (20 subfamilies). Nineteen of them are present in Porifera and fourteen in Choanoflagelida and Filasteria (the closest unicellular relatives of animal). The analysis supports the idea that Dbl-like RhoGEFs were present at the origin of eukaryotes and, in the course of evolution, developed as very adaptive cell signaling mediators. In non-bilaterian metazoans, Fort and Biangly identified 28, 22, and 26 RhoGEF members in the genomes of Cnidaria, Placozoa, and Porifera, respectively [15]. Since Porifera is one of the earliest-branching metazoan lineages, it is very likely that the ancestral Metazoa had at least 19 of 20 RhoGEF vertebrate subfamilies. This implies that the molecular pathways controlled by RhoGEF subfamilies were already established at the origin of Metazoa [15].

## 4. Conclusions

Based on current knowledge on Rho-family GTPases in non-bilaterian animals, the available experimental data about their biochemical characteristics and functions as well as our bioinformatics analysis, we confirmed the presence of Rho and Cdc42 homologs in the genomes of all basal Metazoa. A member of the Rac family was found in the genomes of Porifera, Placozoa, and Cnidaria, while a member of the Miro family is present in the genomes of Cnidaria and Placozoa. Members of Rnd, RhoUV, and RhoBTB families are present in the genomes of Cnidaria. These findings support the conclusion that the ancestor of all animals probably contained Rho, Rac, and Cdc42 homologs. We failed to confirm the Rac gene in the currently available placozoan genome database. This could be due to gene loss, incomplete and/or low quality genome assemblies, and/or gene annotation. RhoUV and Rnd genes probably emerged before the divergence of Cnidaria. A varying number of paralogs in early-branching animals is probably a consequence of lineage-specific duplications or gene loss. Protein analysis revealed that Rho, Rac, Cdc42, RhoBTB, and Miro homologs present in basal Metazoa show high similarity in primary and secondary structures with homologs in “higher” metazoans, implying possible similar or identical biochemical and biological functions. Therefore, we believe that basal metazoans represent an important model for studying the properties of ancestral metazoan homologs before their diversification in higher animals. Moreover, the use of these animals as experimental models is imperative for understanding the causes, biology, and prevention of human diseases, especially cancer.

## Figures and Tables

**Table 1:** Rho GTPases in Metazoa and close unicellular relative of Metazoa.

Taxonomic Groups		Organism	RHO GTPases								
			*Rho*	*Rac*	*Cdc42*	*Rnd*	*RhoDF*	*RhoUV*	*RhoH*	*RhoBTB*	*Miro*
**Bilateria**	Mammalia	*Homo sapiens*	*HsRhoA* *HsRhoB* *HsRhoC* *HsRhoG* *HsRhoJ* *HsRhoQ*	*HsRac1* *HsRac2* *HsRac3*	*HsCdc42*	*HsRnd1* *HsRnd2* *HsRnd3*	*Hsrhod* *HsRhoF*	*HsRhoU* *HsRhoV*	*HsRhoH*	*HsRhoBTB1* *HsRhoBTB2*	*HsMiro1* *HsMiro2*
	Aves	*G. galus*	*GgRhoA* *GgRhoB* *GgRhoC* *GgRhoG* *GgRhoJ* *GgRhoQ*	*GgRac1* *GgRac2* *GgRac3*	*GgCdc42*	*GgRnd1* *GgRnd2* *GgRnd3*	*GgRhoD* *GgRhoF*	*GgRhoU* *GgRhoV*	*GgRhoH*	*Gg* *RhoBTB1* *Gg* *RhoBTB2*	*GgMiro1* *GgMiro2*
	Amphibia	*X. tropicallis*	*XtRhoA* *XtRhoB* *XtRhoC* *XtRhoG* *XtRhoJ* *XtRhoQ*	*XtRac1* *XtRac2* *XtRac3*	*XtCdc42*	*XtRnd1* *XtRnd3*	*XtRhoF*	*XtRhoU* *XtRhoV*	*XtRhoH*	*Xt* *RhoBTB1* *Xt* *RhoBTB2*	*XtMiro1* *XtMiro2*
	Chordata	*D. rerio*	*DrRhoA* *DrRhoC* *DrRhoG* *DrRhoJ* *DrRhoQ*	*DrRac1* *DrRac2* *DrRac3*	*DrCdc42*	*DrRnd1* *DrRnd2* *DrRnd3*	*DrRhoF*	*DrRhoU* *DrRhoV*	*DrRhoH*	*Dr* *RhoBTB1* *Dr* *RhoBTB2*	*DrMiro1* *DrMiro2*
	Cephalochordata	*B. floridae*	*BfRho1-1* *BfRho1-2* *BfTc10/RhoQ* *BfTc10/RhoQ* *BfRhoA* *BfRhoC*	*BfRac1* *BfRac2* *BfRac3* *BfRac4*	*BfCdc42*	*BfRnd*	*BfRif*	*BfRhoU*		*BfRhoBTB*	*BfMiro*
	Echinodermata	*S. purpuratus*	*SpRho1* *RhoA-A* *SpTc10/RhoQ* *SpRhoA* *SpRhoC*	*SpRac1* *SpRacB* *SpRac2* *SpRac3* *SpRac4*	*SpCdc42*			*SpRhoU*		*SpRhoBTB*	*SpMiro*
	Arthropoda	*D. melanogaster*	*DmRho*	*DmRac1* *DmRac2*	*DmCdc42*					*DmRhoBTB*	*DmMiro*
	Nematoda	*C. elegans*	*CeRho1*	*CeRac * *CeRac2* *CeCed10*	*CeCdc42*						*CeMiro1* *CeMiro2*
	Mollusca	*C. gigas*	*CgRho1* *CgRho2* *CgRhoJ* *RhoQ*	*CgRac1* *CgRac2*	*CgCdc42*	*CgRnd*		*CgRhoU*		*CgRhoBTB*	*CgMiro*
**Non-Bilateria**	Cnidaria	*N. vectensis*	*NvRho* *NvRho2*	*NvRac1*	*NvCdc42*	*NvRnd3*		*NvRhoU*		*NvRhoBTB1*	*NvMiro*
		*H. vulgaris*	*HvRho1* *HvRho2* *HvRho3*	*HvRac1*	*HvCdc42*	*HvRnd3*				*HvRhoBTB1*	*HvMiro*
	Placozoa	*T. adherens*	*TaRhoA*	*TaRac1*	*TaCdc42*						*TaMiro*
	Ctenophora	*M. leidyi*	*MlRho*		*MlCdc42*						
	Porifera	*S. domuncula*	*SdRho1* *SdRhoA* *SdRho3*	*SdRac*	*SdCdc42*						
		*A. queenslandica*	*AqRho* *AqRhoA*	*AqRac1* *AqRac2*	*AqCdc42*						
	Protista	*M. brevicoliis*	*MbRho*	*MbRac*	*MbCdc42*						

**Table 2:** Rho GTPases from basal Metazoa: comparison with most related human proteins.

Basal Metazoa				*Homo Sapiens*			
Taxonomic Group	Organism	Name	No. aa	Name	No. aa	ident%	simil%
**Cnidaria**	*N. vectensis*	NvRho NvRho2 NvRac1 NvCdc42 NvRhoBTB NvMiro NvRhoU NvRnd3	192 214 194 191 660 662 211 163	RhoA RhoA Rac1 Cdc42 BTB1 Miro1 RhoV Rnd3	193 193 192 191 696 618 236 244	89.1 43 89.2 91 37.7 53.7 54 35	94.3 60.7 93.3 95.3 54 70.1 59 50
	*H. vulgaris*	HvRho1 HvRho2 HvRho3 HvRac1 HvuRnd3 HvCdc42 HvRhoBTB HvMiro	192 192 211 192 194 191 679 622	RhoA RhoA RhoA Rac1 Rnd3 Cdc42 BTB1 Miro1	193 193 193 192 244 191 696 618	88.1 87.6 54.5 89.1 28 89.5 33.8 45.6	94.3 92.2 71.1 92.2 46 94.8 51.7 65.8
**Placozoa**	*T. adherens*	TaRhoA TaRac1 TaCdc42 TaMiro	193 197 187 586	RhoA Rac1 Cdc42 Miro1	193 192 191 618	88.1 79.7 86.4 50.2	93.8 87.3 92.7 66
**Ctenophora**	*M. leidyi*	MlRho MlCdc42	192 192	RhoA Cdc42	193 191	86.5 8.5	91.2 94.3
**Porifera**	*S. domuncula*	SdRho1 SdRhoA SdRho3 SdRac SdCdc42	192 192 195 192 191	RhoA RhoB RhoA Rac1 Cdc42	193 196 193 192 191	86 71.4 68.2 70.3 89.5	91.7 83.7 79 83.3 94.8
	*A. queenslandica*	AqRho AqRhoA AqRac1 AqRac2 AqCdc42	192 193 192 201 191	RhoA RhoA Rac1 Rac1 Cdc42	193 193 192 192 191	85 34.9 88.5 68.7 90.6	90.7 53.9 94.3 81.6 94.8

## Supplementary Materials

The following are available online at https://www.mdpi.com/2073-4409/9/10/2279/s1, Table S1: Selected metazoan species accession numbers.
